# Recommendations of the Advisory Committee on Immunization Practices for Use of Herpes Zoster Vaccines

**DOI:** 10.15585/mmwr.mm6703a5

**Published:** 2018-01-26

**Authors:** Kathleen L. Dooling, Angela Guo, Manisha Patel, Grace M. Lee, Kelly Moore, Edward A. Belongia, Rafael Harpaz

**Affiliations:** ^1^Division of Viral Diseases, National Center for Immunization and Respiratory Disease, CDC; ^2^Stanford University School of Medicine, Stanford, California; ^3^Tennessee Department of Health; ^4^Marshfield Clinic Research Institute, Marshfield, Wisconsin.

## Introduction

On October 20, 2017, Zoster Vaccine Recombinant, Adjuvanted (Shingrix, GlaxoSmithKline, [GSK] Research Triangle Park, North Carolina), a 2-dose, subunit vaccine containing recombinant glycoprotein E in combination with a novel adjuvant (AS01_B_), was approved by the Food and Drug Administration for the prevention of herpes zoster in adults aged ≥50 years. The vaccine consists of 2 doses (0.5 mL each), administered intramuscularly, 2–6 months apart (*1*). On October 25, 2017, the Advisory Committee on Immunization Practices (ACIP) recommended the recombinant zoster vaccine (RZV) for use in immunocompetent adults aged ≥50 years.

Herpes zoster is a localized, usually painful, cutaneous eruption resulting from reactivation of latent varicella zoster virus (VZV). Herpes zoster is common: approximately one million cases occur each year in the United States (*2*). The incidence increases with age, from five cases per 1,000 population in adults aged 50–59 years to 11 cases per 1,000 population in persons aged ≥80 years (*2*). Postherpetic neuralgia, commonly defined as persistent pain for at least 90 days following the resolution of the herpes zoster rash, is the most common complication and occurs in 10%–13% of herpes zoster cases in persons aged >50 years (*3*,*4*). Among persons with herpes zoster, the risk for developing postherpetic neuralgia also increases with age (*3*–*5*).

Zoster Vaccine Live (ZVL) (Zostavax, Merck and Co., Inc., Whitehouse Station, New Jersey), a 1-dose live attenuated strain of VZV, is licensed for the prevention of herpes zoster in immunocompetent adults aged ≥50 years and is recommended by the ACIP for use in immunocompetent adults aged ≥60 years (*6*). Since licensure, vaccine coverage has increased each year, and by 2016, 33% of adults aged ≥60 years reported receipt of the vaccine (CDC, provisional unpublished data). ACIP considered use of RZV, as well as existing recommendations, to develop vaccination policy which would be safe and reduce disease burden. This report serves as a supplement to the 2008 Prevention of Herpes Zoster Recommendations of ACIP for the use of ZVL in adults aged ≥60 years and subsequent updates (*6*–*8*); it outlines recent ACIP recommendations as well as guidance for use of RZV and ZVL in adults.

## Methods

From March 2015 to October 2017, the ACIP Herpes Zoster Vaccines Work Group (Work Group; see acknowledgments for members and their affiliations) participated in monthly or bimonthly teleconferences to review herpes zoster epidemiology and the evidence for the efficacy, safety, and programmatic factors of RZV and ZVL. According to the Grading of Recommendations Assessment, Development and Evaluation (GRADE) approach, the Work Group defined critical and important outcomes, conducted a systematic review of the evidence, and subsequently reviewed and discussed findings and evidence quality (https://www.cdc.gov/vaccines/acip/recs/grade/) (*9*).

A cost effectiveness analysis comparing RZV, ZVL, or no vaccine was conducted by CDC from a societal perspective, using an analytic horizon of time of vaccination through the end of life. Model inputs were based on published literature where available and relied on unpublished data and Work Group expert opinion when necessary. It was modeled that ZVL effectiveness against herpes zoster would wane to zero 4–12 years following vaccination, depending on age at vaccination (*4*,*10*–*13*). In the absence of long-term effectiveness data, it was modeled that RZV effectiveness in adults aged 50–69 years or ≥70 years would wane to zero 19 years following vaccination based on the rate of waning observed during the first 4 years of clinical trials as well as expert opinion (*13*–*15*). Economic analyses were also conducted for RZV in cohorts previously vaccinated with ZVL. In keeping with CDC practice (*16*,*17*), the purpose of the economic analysis was to model the proposed recommendation; therefore, full adherence to a 2-dose RZV regime was assumed in baseline models. Lower rates of 2-dose adherence were evaluated in sensitivity analyses.

Since 2015, RZV was discussed at five ACIP meetings. In addition to the aforementioned data, several independent health economic studies (*18*,*19*), (Merck, unpublished data, 2017), as well as immunogenicity data were presented. Long-term immunogenicity of RZV (*20*) and immunogenicity and safety of RZV in ZVL recipients (*21*) were considered, with recognition that there are no standard immunologic correlates of protection for prevention of herpes zoster.

At the October 2017 meeting, three proposed recommendations were presented to the committee, and, after a public comment period, were approved by the voting ACIP members as follows: 1) RZV is recommended for immunocompetent adults aged ≥50 years (14 voted in favor, 1 opposed*), 2) RZV is recommended for immunocompetent adults previously vaccinated with ZVL (12 voted in favor, 3 opposed), and 3) RZV is preferred over ZVL (8 voted in favor, 7 opposed). This report summarizes the data considered, the quality of evidence, and rationale for recommendations.

## Summary of Findings

As a result of the GRADE process, key outcomes were designated as critical (prevention of herpes zoster and postherpetic neuralgia, serious adverse events following vaccination) or important (duration of protection, reactogenicity). All outcomes were considered for both RZV and ZVL compared with no vaccination. There were no clinical studies that compared the vaccines directly with one another (head-to-head). Supporting evidence for the Work Group’s findings is available online (https://www.cdc.gov/vaccines/acip/recs/grade/herpes-zoster.html) (*22*).

**Recombinant Zoster Vaccine (RZV)**. Efficacy of RZV was evaluated in a two-part, phase III multicenter clinical trial which enrolled >30,000 participants, who were randomized 1:1 to receive vaccine or saline placebo (*14*,*15*). The median follow-up time was 3.2 years for Zoster Efficacy Study in Adults 50 Years of Age or Older (ZOE-50) (*14*), and 3.7 years for Zoster Efficacy Study in Adults 70 Years of Age or Older (ZOE-70) (*15*). The efficacy for the prevention of herpes zoster was 96.6% (95% confidence interval [CI] = 89.6–99.3) in persons aged 50–59 years and 97.4% (95% CI = 90.1–99.7) in persons aged 60–69 years (*14*). Using pooled data from both study arms, vaccine efficacy was 91.3% (95% CI = 86.8–94.5) in participants aged ≥70 years (*15*). Vaccine efficacy in the first year after vaccination was 97.6% (95% CI = 90.9–99.8) and was 84.7% (95% CI = 69.0–93.4) or higher for the remaining 3 years of the study in persons aged ≥70 years. Efficacy for prevention of postherpetic neuralgia was 91.2% (95% CI = 75.9–97.7) in adults aged ≥50 years and 88.8% (95% CI = 68.7–97.1) in those aged ≥70 years (*15*).

Serious adverse events (an undesirable experience associated with the vaccine that results in death, hospitalization, disability or requires medical or surgical intervention to prevent a serious outcome) were examined in eight studies sponsored by GSK, which included 29,965 subjects (15,264 RZV recipients) (*22*). Overall, rates of serious adverse events over the study periods were similar in the RZV and placebo groups.

Injection-site and systemic grade 3 solicited adverse events (reactions related to vaccination which were severe enough to prevent normal activities) were actively surveyed in eight studies involving 10,590 subjects (*22*). Among the subset of subjects completing the 7-day diary card for reactogenicity in phase III clinical trials (9,936), 16.5% of vaccine recipients reported any grade 3 adverse event compared with 3.1% of placebo recipients (*14*,*15*). Grade 3 injection-site reactions (pain, redness, and swelling) were reported by 9.4% of vaccine recipients, compared with 0.3% of placebo recipients and grade 3 solicited systemic events (myalgia, fatigue, headache, shivering, fever, and gastrointestinal symptoms) were reported by 10.8% of vaccine recipients and 2.4% of placebo recipients (*14**,**15*). Whereas there were no differences in the proportions of local grade 3 reactions between dose 1 and dose 2, systemic grade 3 reactions were reported more frequently after dose 2 (*1*). Overall, the most common solicited adverse reactions (grade 1–3) were pain (78%), myalgia (45%), and fatigue (45%) (*1*).

**Zoster Vaccine Live (ZVL)**. Two randomized clinical trials and seven observational studies were reviewed to evaluate the performance of a single dose of ZVL in preventing herpes zoster (*22*). A randomized clinical trial in persons aged 50–59 years found that the efficacy was 70% (95% CI = 54–81) (median follow-up time was 1.3 years) (*12*). A randomized trial in persons aged ≥60 years found that the efficacy was 64% (95% CI = 56–71) in persons aged 60–69 years and 38% (95% CI = 25–48) in persons aged ≥70 (median follow-up time was 3.1 years) (*4*). Estimates from observational studies and randomized controlled trials (RCTs) are consistent; observational estimates are within the 95% CI of the RCT estimates (*22*). The duration of protection has been studied out to 11 years, including the first 4 years of the RCT and then follow-on, nonblinded studies which used a modeled control group from years 7–11 (*4*,*10*,*11*). Shorter follow-up periods have been evaluated in observational studies using administrative health data (*22*). Studies concur that there is a substantial decrease in effectiveness following the first year after receipt of ZVL, and, by 6 years postvaccination, vaccine effectiveness against herpes zoster is <35% (*10*,*23*–*25*). During years 7–8 postvaccination, observational study estimates of effectiveness ranged from 21%–32% (*23**,**24*). In the longest study of ZVL, estimates of effectiveness were no longer statistically significant 9–11 years postvaccination (*11*). In a phase III clinical trial, vaccine efficacy against post herpetic neuralgia was 65.7% (95% CI = 20.4–86.7) in persons aged 60–69 years and 66.8% (95% CI = 43.3–81.3) in participants aged ≥70 years (median follow-up of 3.1 years) (*4*); these estimates are consistent with estimates from observational studies (*22*). Notably, in observational studies, vaccine effectiveness against postherpetic neuralgia was longer-lasting than effectiveness against herpes zoster itself (*23*,*26*).

Serious adverse events related to ZVL were examined in eight high quality RCTs, 13 RCTs with limitations, and an additional seven observational studies (*22*). Overall, serious adverse events occurred at similar rates in vaccinated and placebo groups. Whereas injection site reactions were reported in 48% of vaccine recipients and 17% of placebo recipients in phase III clinical trials, post hoc analysis indicates that no more than 0.9% of vaccine recipients reported any given injection site symptom as grade 3 (*22*). In addition, in rare instances, ZVL vaccine strain has been documented to cause disseminated rash as well as herpes zoster in immunocompetent recipients (*22*,*27*), and life-threatening and fatal complications in immunocompromised recipients (*28*,*29*).

**Cost effectiveness.** The CDC analysis was conducted from a societal perspective over a lifetime. It estimated that vaccination with RZV, compared with no vaccination, cost $31,000 per quality adjusted life year (QALY), on average, for immunocompetent adults aged ≥50 years. The numbers of persons needed to be vaccinated with RZV to prevent one case of herpes zoster and one case of postherpetic neuralgia are 11–17 and 70–187, respectively. Estimates of costs per QALY for vaccination with RZV 8 weeks following ZVL (estimated by immediate revaccination in the model) ranged from $15,000 per QALY in persons aged 80–89 years to $117,000 per QALY for persons aged 50–59 years. Under most assumptions, vaccination with RZV prevented more disease at lower overall costs than did vaccination with ZVL. In probabilistic sensitivity analyses, 73.5% 2-dose completion (range = 38.8%–96.3%) coupled with 1-dose initial effectiveness estimates of 90% and 69% were applied, and RZV remained the most cost-effective strategy (*13*).

ACIP also reviewed independent cost-effectiveness analyses by an academic group (*18*), GSK (*19*), and Merck (Merck, unpublished data, 2017). The academic group estimated RZV costs per QALY of $30,000 when vaccination occurred at age 60 years. The GSK model estimated RZV costs per QALY of $12,000, on average, for recipients aged ≥60 years. Although analytic approaches and model inputs differed, both groups found that RZV was more cost effective than ZVL. Merck modeled vaccination at age ≥60 years and estimated $107,000 per QALY for RZV and $83,000 per QALY for ZVL, with ZVL as the most cost-effective vaccine in most scenarios.

## Summary of the Quality of Evidence Across Outcomes

The body of evidence for benefits of RZV (prevention of herpes zoster and postherpetic neuralgia and duration of protection against herpes zoster) was primarily informed by one high quality RCT that studied vaccine efficacy through 4 years postvaccination. The GRADE evidence type was judged as 1, the strongest level of evidence (*22*). The evidence for possible harms (serious adverse events and reactogenicity) was reported in the same RCT and was consistent across additional smaller, less rigorous studies. Overall, the estimates of possible harms were supported by GRADE evidence type 1 (*22*).

The body of evidence for benefits of ZVL (prevention of herpes zoster and postherpetic neuralgia, and duration of protection against herpes zoster) was large, including a high quality prelicensure RCT as well as a postlicensure RCT and observational studies of effectiveness. The level of vaccine effectiveness for the prevention of herpes zoster and postherpetic neuralgia was supported by GRADE evidence type 1 (*22*). The duration of protection beyond 4 years was supported by GRADE evidence type 2 because the studies lacked blinding, and beyond 6 years, lacked randomization and a true control group. The evidence for possible harms of ZVL (serious adverse events and reactogenicity) was supported by GRADE evidence type 1 from multiple RCTs and supported by observational studies and a decade of experience (*22*,*29*).

## Rationale

**RZV use in immunocompetent adults aged ≥50 years**. With high efficacy among adults aged ≥50 years, and modest waning of protection over 4 years following vaccination, RZV has the potential to prevent substantial herpes zoster disease burden. Vaccinating adults starting at age 50 will prevent disease incidence in midlife, and the vaccine will likely continue to provide substantial protection beyond 4 years as recipients age.

**RZV use in immunocompetent adults who previously received ZVL**. In separate clinical trials, RZV estimates of efficacy against herpes zoster were higher than ZVL estimates in all age categories. The difference in efficacy between the two vaccines was most pronounced among recipients aged ≥70 years. Studies have shown that ZVL effectiveness wanes substantially over time, leaving recipients with reduced protection against herpes zoster. RZV elicited similar safety, reactogenicity, and immunogenicity profiles regardless of prior ZVL receipt; therefore, ZVL recipients will likely benefit from vaccination with RZV.

**Preferential use of RZV**. In separate clinical trials, for all age categories, RZV estimates of efficacy against herpes zoster were higher than those for ZVL. Estimates of efficacy against postherpetic neuralgia are also higher for RZV than for ZVL; however, CIs overlap. ZVL efficacy wanes substantially during the 4 years following receipt. As a result of higher and more long-lasting efficacy, RZV is estimated to prevent more herpes zoster and postherpetic neuralgia compared with ZVL. ACIP acknowledged that several aspects of RZV performance will be further elucidated postlicensure, including the possibility of a rare adverse event related to the vaccine, the long-term duration of protection, the adherence to the 2-dose schedule, and the effectiveness and duration of protection of 1 dose of RZV. Some ACIP members preferred to recommend both vaccines with no preference until real-world data could be accrued, including head-to-head studies. The majority of ACIP members voted to recommend RZV preferentially (Box).

BOX **Recommendations for the use of herpes zoster vaccines**

In October 2017, the Advisory Committee on Immunization Practices (ACIP) made the following three recommendations:

1. Recombinant zoster vaccine (RZV) is recommended for the prevention of herpes zoster and related complications for immunocompetent adults aged ≥50 years.
2. RZV is recommended for the prevention of herpes zoster and related complications for immunocompetent adults who previously received zoster vaccine live (ZVL).
3. RZV is preferred over ZVL for the prevention of herpes zoster and related complications.

These recommendations serve as a supplement to the existing recommendations for the use of ZVL in immunocompetent adults aged ≥60 years.

## Clinical Guidance

**General use**. RZV may be used in adults aged ≥50 years, irrespective of prior receipt of varicella vaccine or ZVL, and does not require screening for a history of chickenpox (varicella). ZVL remains a recommended vaccine for prevention of herpes zoster in immunocompetent adults aged ≥60 years (*6*). Care should be taken not to confuse ZVL, which is stored in the freezer and administered subcutaneously, with RZV, which is stored in the refrigerator and administered intramuscularly.

**Dosing schedule.** Following the first dose of RZV, the second dose should be given 2–6 months later (*1*). The vaccine series need not be restarted if more than 6 months have elapsed since the first dose; however, the efficacy of alternative dosing regimens has not been evaluated, data regarding the safety of alternative regimens are limited (*30*), and individuals might remain at risk for herpes zoster during a longer than recommended interval between doses 1 and 2. If the second dose of RZV is given less than 4 weeks after the first, the second dose should be repeated. Two doses of the vaccine are necessary regardless of prior history of herpes zoster or prior receipt of ZVL.

**Timing of RZV for persons previously vaccinated with ZVL**. Age and time since receipt of ZVL may be considered to determine when to vaccinate with RZV. Studies examined the safety and immunogenicity of RZV vaccination administered ≥5 years after ZVL (*21*); shorter intervals have not been studied. However, there are no data or theoretical concerns to indicate that RZV would be less safe or less effective when administered at an interval of <5 years. Clinical trials indicated lower efficacy of ZVL in adults aged ≥70 years; therefore, a shorter interval may be considered based on the recipient’s age when ZVL was administered. Based on expert opinion, RZV should not be given <2 months after receipt of ZVL.

**Coadministration with other vaccines.** CDC’s general best practice guidelines for immunization advise that recombinant and adjuvanted vaccines, such as RZV, can be administered concomitantly, at different anatomic sites, with other adult vaccines (*31*). Concomitant administration of RZV with Fluarix Quadrivalent (influenza vaccine) (QIV) has been studied, and there was no evidence for interference in the immune response to either vaccine or safety concerns (*32*). Evaluation of coadministration of RZV with 23-valent pneumococcal polysaccharide vaccine (PPSV23, Pneumovax23) and tetanus toxoid, reduced diphtheria toxoid, and acellular pertussis vaccine, adsorbed (Tdap, Boostrix) is ongoing. The safety and efficacy of administration of two adjuvanted vaccines (e.g., RZV and adjuvanted influenza vaccine [Fluad]), either concomitantly or at other intervals, have not been evaluated.

**Counseling for reactogenicity.** Before vaccination, providers should counsel RZV recipients about expected systemic and local reactogenicity. Reactions to the first dose did not strongly predict reactions to the second dose (*33*); vaccine recipients should be encouraged to complete the series even if they experienced a grade 1–3 reaction to the first dose of RZV. The impact of prophylactic analgesics in conjunction with RZV has not been studied.

## Special Populations

**Persons with a history of herpes zoster**. Herpes zoster can recur. Adults with a history of herpes zoster should receive RZV. If a patient is experiencing an episode of herpes zoster, vaccination should be delayed until the acute stage of the illness is over and symptoms abate. Studies of safety and immunogenicity of RZV in this population are ongoing.

**Persons with chronic medical conditions.** Adults with chronic medical conditions (e.g., chronic renal failure, diabetes mellitus, rheumatoid arthritis, and chronic pulmonary disease) should receive RZV.

**Immunocompromised persons**. As with ZVL, the ACIP recommends the use of RZV in persons taking low-dose immunosuppressive therapy (e.g., <20 mg/day of prednisone or equivalent or using inhaled or topical steroids) and persons anticipating immunosuppression or who have recovered from an immunocompromising illness (*6*). Whereas RZV is licensed for all persons aged ≥50 years, immunocompromised persons and those on moderate to high doses of immunosuppressive therapy were excluded from the efficacy studies (ZOE-50 and ZOE-70), and thus, ACIP has not made recommendations regarding the use of RZV in these patients; this topic is anticipated to be discussed at upcoming ACIP meetings as additional data become available.

**Persons known to be VZV negative**. Screening for a history of varicella (either verbally or via laboratory serology) before vaccination for herpes zoster is not recommended. However, in persons known to be VZV negative via serologic testing, ACIP guidelines for varicella vaccination should be followed. RZV has not been evaluated in persons who are VZV seronegative and the vaccine is not indicated for the prevention of chickenpox (varicella).

## Contraindication

**Allergy**. RZV should not be administered to persons with a history of severe allergic reaction, such as anaphylaxis, to any component of this vaccine.

## Precautions

**Current herpes zoster infection**. RZV is not a treatment for herpes zoster or postherpetic neuralgia and should not be administered during an acute episode of herpes zoster.

**Pregnancy and breastfeeding**. There are no available data to establish whether RZV is safe in pregnant or lactating women and there is currently no ACIP recommendation for RZV use in this population. Consider delaying vaccination with RZV in such circumstances.

## Reporting of Vaccine Adverse Reactions

Adverse events that occur in a patient following vaccination can be reported to the Vaccine Adverse Events Reporting System (VAERS). Reporting is encouraged for any clinically significant adverse event even if it is uncertain whether the vaccine caused the event. Information on how to submit a report to VAERS is available at https://vaers.hhs.gov/index.html or by telephone at 1–800–822–7967.

## Future Research and Monitoring Priorities

Studies of safety, immunogenicity, and efficacy of herpes zoster vaccines in defined immunocompromised populations are ongoing. ACIP will consider these data as they become available and revise recommendations accordingly. In addition, CDC will monitor coverage of RZV and adherence to the 2-dose schedule. Short-term and long-term effectiveness of RZV will be assessed through longitudinal studies of clinical trial participants as well as through observational studies.

As with all new vaccines, CDC will monitor adverse events following immunization through VAERS and the Vaccine Safety Datalink. Additional post-marketing safety monitoring will include studies conducted by GSK and reported to the FDA. Monitoring RZV is particularly important given the vaccine’s novel adjuvant and its high reactogenicity and immunogenicity.

## Footnotes

* Laura Riley submitted that her opposed vote was cast in error. This is reflected in the official minutes; however, because the disclosure occurred after the session was closed, the original vote remains unchanged.
